# Protegrin-1 cytotoxicity towards mammalian cells positively correlates with the magnitude of conformational changes of the unfolded form upon cell interaction

**DOI:** 10.1038/s41598-019-47955-2

**Published:** 2019-08-09

**Authors:** Nagasundarapandian Soundrarajan, Suhyun Park, Quy Le Van Chanh, Hye-sun Cho, Govindan Raghunathan, Byeongyong Ahn, Hyuk Song, Jin-Hoi Kim, Chankyu Park

**Affiliations:** 1 Department of Stem Cell and Regenerative Biotechnology, Konkuk University, Gwangjin-gu, Seoul, South Korea; 2 Department of Chemistry, University of Konstanz, Universitӓtsstraße 10, 78457 Konstanz, Germany

**Keywords:** Antimicrobial responses, Biological fluorescence

## Abstract

Porcine protegrin-1 (PG-1) is a broad-spectrum antimicrobial peptide (AMP) with potent antimicrobial activities. We produced recombinant PG-1 and evaluated its cytotoxicity toward various types of mammalian cell lines, including embryonic fibroblasts, retinal cells, embryonic kidney cells, neuroblastoma cells, alveolar macrophage cells, and neutrophils. The sensitivity of the different mammalian cells to cytotoxic damage induced by PG-1 differed significantly among the cell types, with retinal neuron cells and neutrophils being the most significantly affected. A circular dichroism analysis showed there was a precise correlation between conformational changes in PG-1 and the magnitude of cytotoxicity among the various cell type. Subsequently, a green fluorescent protein (GFP) penetration assay using positively charged GFPs indicated there was a close correlation between the degree of penetration of charged GFP into cells and the magnitude of PG-1 cytotoxicity. Furthermore, we also showed that inhibition of the synthesis of anionic sulphated proteoglycans on the cell surface decreases the cytotoxic damage induced by PG-1 treatment. Taken together, the observed cytotoxicity of PG-1 towards different membrane surfaces is highly driven by the membrane’s anionic properties. Our results reveal a possible mechanism underlying cell-type dependent differences in cytotoxicity of AMPs, such as PG-1, toward mammalian cells.

## Introduction

Antimicrobial peptides (AMPs) are small charged molecules that are involved in the innate immune system, and whose primary function is to eliminate invading pathogens^1,2^. They evoke activity against pathogenic bacteria by forming pores in the membrane, by inhibiting key cellular mechanisms such as translation/transcription after penetrating the cytoplasm, or by bacterial agglutination^3–6^. Recently, the growing incidence of antibiotic resistant bacteria has become a big concern in the health care industry^7^, and AMPs are being exploited as alternatives or combinatorial candidates with antibiotics to combat such pathogens^8,9^. The availability of genome sequences from diverse species also provides increased opportunities for the discovery of new AMPs^10,11^.

The more negatively charged and cholesterol-poor structure of bacterial membranes result in stronger binding of AMPs compared to their mammalian counterparts. The presence of cholesterol in the mammalian membrane also weakens the hydrophobic interactions with the AMPs^12,13^. However, AMPs also show varying degrees of cytotoxicity towards mammalian cells^14^. The use of AMPs for clinical applications raises concerns about the cytotoxic effects of AMPs in humans. The therapeutic index (TI) is the ratio between the minimal inhibitory concentration (MIC) towards pathogenic microorganisms and the concentration that causes erythrocyte haemolysis. Thus, larger TI values are required for drug candidates^15^.

**Figure 1:**
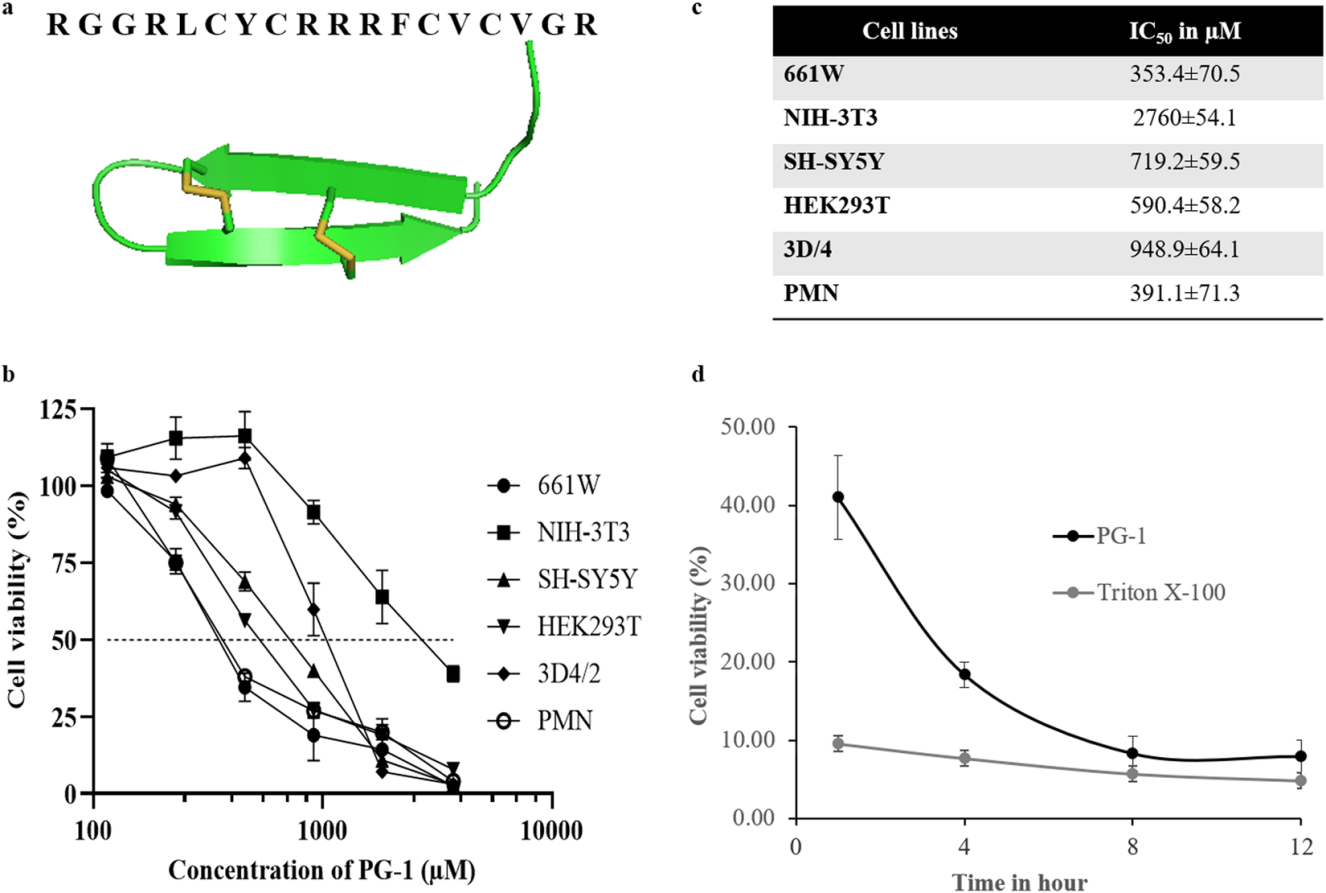
Cytotoxic profile of protegrin-1 to various mammalian cells. (**a**) Three-dimensional structure of PG-1 and its amino acid sequence are shown. The structure was created using the PBD ID 1PG1 using Pymol. (**b**) Cytotoxicity was measured as decreases in cell viability. 661W, HEK293T, NIH-3T3, SH-SY5Y, PMN, and 3D4/2 cells were treated with various concentrations of recombinant PG-1 and their viability were measured after 24 h of treatment. (**c**) IC_50_ of PG-1 for different cell types after 24 h exposure. IC_50_, minimum concentration required to inhibit 50% of cell growth. The results were presented with 95% confidence interval. (**d**) 661W cells were treated with 6600 µM of PG-1 and viability were measured at various time intervals (1, 4, 8, and 12 h). Triton X-100 and PBS were used as the positive and negative controls, respectively. The experiments were carried out in triplicates. Cell viability was presented as mean ± standard deviation (**b**,**c**).

Protegrin-1 (PG-1) is a small cationic AMP (CAP) that contains a β-hairpin structure stabilized by disulphide bonds (Fig. 1a) and shows a strong antimicrobial activity against a board spectrum of pathogens including multi-drug resistance bacteria^16^. PG-1 belongs to the cathelicidin family and was first identified in porcine neutrophils. Five different allelic forms of PG-1 have been reported^17^.

The cytotoxic activities produced by AMPs are caused by multiple individual mechanisms, or a combination of mechanisms, that cause cell lysis leading to necrosis or programmed cell death^18–20^. Numerous studies were conducted using α-helical AMPs to elucidate the mechanisms of cytotoxicity, which generally caused by higher threshold of hydrophobicity, helical propensity and the composition of anionic headgroup components like PG or PS (Phosphatidyl-*rac*-glycerol or serine, respectively) present in the mammalian membranes^21–23^. Some CAPs including PG-1, tachyplesin-1, and polyphemusin-1 show high degrees of haemolytic and cytotoxic activities toward mammalian cells compared with other AMPs^18,24–26^. Several studies experimentally as well as computationally showed that PG-1 enters the bilipid layer by making toroidal pores which vastly depend on their charges, amphipathicity, folding into β-hairpin, and stability of β-barrel pore-forming by oligomerization^3,26–29^. The spider peptide gomesin shows a specific cytotoxicity towards neuroblastoma and cancer cells without displaying any significant cytotoxicity to erythrocytes and Hep G2 cells, indicating there are differences in cytotoxicity depending on the cell type^19,26,30^. However, a systemic evaluation of the varied cytotoxic effect of AMPs in different mammalian cells and their mechanisms has not been well understood for β-sheet AMPs.

In this study, we produced a recombinant form of PG-1^31^ and evaluated differences in cytotoxic damage toward different types of mammalian cells after PG-1 treatment. Our results showed significant differences in PG-1 cytotoxicity in different cell types. To investigate the underlying mechanisms associated with these variations, using circular dichroism (CD) we analysed differences in peptide conformation when in the presence of the different cell types. We also used a super charged-GFP penetration assay in the different cell types and assessed the effect of reducing the levels of cellular anionic sulphated proteoglycans on uptake. From these analyses, we identified factors that influence mammalian cell cytotoxicity to cathelicidins such as PG-1 and suggest that the level of cytotoxicity is influenced by multiple factors including the magnitude of the membrane charge on the cell surface and the conformational changes that occur in PG-1 upon cell interaction.

## Results

### Successful production of recombinant PG-1 in *Escherichia coli*

PG-1 was expressed by transforming the pET30b-based plasmid r5M-172-PG-1-173 into BL21 cells (S1) and cultured in 1 L of Luria broth. The expressed peptide was then purified, refolded, and analysed by electrophoresis on a 16% Tris-tricine SDS-PAGE and peptide was confirmed by probing with rabbit anti-PG-1 antibody (S2). The purified PG-1 showed more than 95% purity assessed by calculating the target peak area of RP-HPLC (S3). A total of 15.4 mg of purified recombinant PG-1 was obtained. The MIC value for the recombinant PG-1 was 3.0 μg/mL against *Escherichia coli* ATCC 25377 which is similar to that of chemically synthesized PG-1^20^.

### Retinal neurons (661W) and neutrophils are susceptible to PG-1 cytotoxicity

The mechanisms underlying the variations in cytotoxic sensitivity among different types of mammalian cells are not clearly understood. We compared the magnitude of the cytotoxic effect from PG-1 exposure using a panel of mammalian cells including 661W, NIH-3T3, SH-SY5Y, 3D4/2, HEK293T, and PMN cells (neutrophils) by evaluating their viability after PG-1 treatment (Fig. 1b). Compared with the other cell types, viability was the lowest in the 661W and PMN cells at 455 µM PG-1, with an almost 3-fold greater reduction in viability over NIH-3T3 and 3D4/2 cells for whom the survival rate was not affected at this concentration. In contrast, PG-1 treatment of SH-SY5Y and HEK293T cells showed an intermediate level of reduction in cell viability. Increases in the PG-1 concentration to 910 and 1365 µM resulted in further decreases in cell viability. The estimated IC_50_ of 661W showed 2 to 7-fold lower than other cell types except PMN (Fig. 1c), which also positively correlates with their cell viability (Fig. 1b). Interestingly, both 661W and SH-SY5Y were neuron cells but 661W showed much lower IC_50_ than that of SH-SY5Y. Therefore, our results show that the cytotoxic activities of PG-1 vary significantly depending on the cell type.

To explore this in more detail, we evaluated changes in cell viability in 661W cells treated with 1365 µM PG-1 at 4 h intervals for a total of 12 h (Fig. 1d). Significant cytotoxicity was evident even at 1 h post-treatment with only 40% of viable cells remaining at this time and the values decreased rapidly to <20% at 4 h post-treatment. The cytotoxicity value approached that of the detergent Triton X-100 at 8 h post treatment. This result was further confirmed by counting the dead and live cells using trypan blue staining (Table ST1) in which the frequencies of unstained viable cells were 8.3% and 0% at 4 and 8 h, respectively. The cytotoxicity of Triton X-100 in MTT assay (Fig. 1d) resulted in 5~10% survivability which is likely to be false positive values triggered by background noise from cell debris or precipitated proteins. Such a bias in the MTT assay has been reported previously^32^.

### The magnitude of AMP folding determines the level of PG-1 cytotoxicity in mammalian cells

Many AMPs are disordered in solution but fold into the proper conformation when they become associated with lipid bilayers^12,33^. However, the magnitude of AMP folding required to reach the final conformations may differ depending on the biochemical characteristics of the associated membranes. As a result, the level of PG-1 cytotoxicity may differ depending on cell types and membrane composition. We analysed the formation of the secondary structure of PG-1 using CD spectroscopy after PG-1 treatment. unfolded PG-1 was also subjected to CD analysis to evaluate differences in secondary structure formation upon association with membranes of different cell types. The MIC value of the unfolded PG-1 was similar to that of folded PG-1, 3 and 4 µg/mL, respectively. The unfolded PG-1 was prepared by DTT treatment and dialysed. The amount of reduced PG-1 after dialysis was determined by derivatization with monobromobiamine. The results showed that about 60 to 70% of reduced PG-1 was present in the 50 to150 µM concentration (S4).

**Figure 2:**
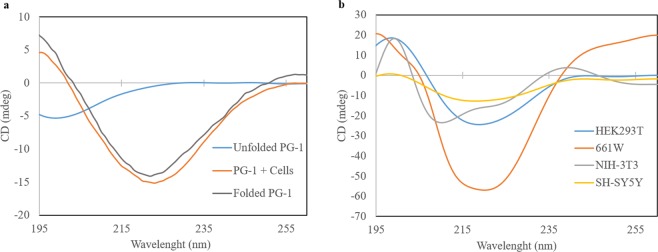
Changes in the structural conformation of PG-1 upon interaction with various cells. (**a**) CD spectra of unfolded PG-1 interacting with *E. coli*. Both folded (shown in grey) and unfolded PG-1 (shown in blue) were suspended in PBS and its CD spectrum were recorded in the absence of cells. *E. coli* cells were incubated with unfolded PG-1 for 2 h and the CD spectrum recorded (shown in orange). The spectra were subtracted from their respective cell spectra without PG-1. (**b**) 661W, NIH-3T3, SH-SY5Y, and HEK293T cells were suspended in PBS and incubated with 150 µM PG-1 for 4 h at 4 °C. The CD spectra for PG-1 in the presence of each cell type is shown in different colours. The spectra were obtained after subtracting the spectra of the respective cells. Representative data from one set of experiments are shown.

The CD spectra of unfolded PG-1 interacting with *E. coli* for 2 h was measured. Comparing to the spectra of unfolded PG-1 in buffer only, a consistent spectral pattern for the β-sheet structure was observed from both folded and *E. coli* interacted PG-1 (Fig. 2a). To obtain the CD spectra arising from the peptide interaction with mammalian cells, we followed the conditions from a previous study^34^. First, an appropriate concentration of PG-1 is required to obtain clear spectra from the peptide interaction with mammalian cells. Therefore, CD spectra were recorded by incubating different concentrations of PG-1 (50, 100, and 150 µM) with the same number of 661W cells for 1 h at 4 °C (Fig. S5). A shallow minimum was most clearly observed at 150 µM PG-1 compared with the other concentrations. To determine the appropriate incubation time, four different cells including 661W were incubated with PG-1 and the spectra were recorded at 0, 1, and 4 h. The most significant difference in the re-folding of PG-1 was observed at 4 h (Fig. S6).

Using the determined parameters, a time course analysis was carried out to observe the folding of PG-1 upon interacting with different cell types. The spectra are shown as subtracted spectra where the spectra from cells incubated in the absence of PG-1 were subtracted from the respective spectra of cells incubated with PG-1. The interaction of PG-1 with 661W cells showed a stronger shallow minimum at 218 nm compared with the other cells (Fig. 2). We also observed a significant difference in the conformational changes in PG-1 upon interacting with the different cell types. Since we were unable to convert the signal mdeg to molar ellipticity, we have presented our data as mdeg vs nm. The spectra shown are representative of a single set of experiments.

Typically, beta sheet containing peptides have a CD spectrum with a negative peak at ~218 nm and a positive peak at ~195 nm. During the time course study, PG-1 interacted with cells and folded slowly to form the proper conformation. However, in 661W cells this folding occurred faster than in other cells in the panel, and thus the CD spectra after 1 h of incubation showed a shallow minimum only in 661W cells (S9). As shown in Figs 2b and S8, the maximum shallow minima were obtained from all the cell types used at 4 h of incubation with PG-1. HEK293T cells showed a shallower minimum at 218 nm compared to NIH-3T3 and SH-SY5Y cells at 4 h of incubation, indicating that the degree of beta-sheet formation is higher than with NIH-3T3 and SH-SY5Y cells. This is consistent with the higher PG-1 cytotoxicity observed in HEK293T cells than in NIH-3T3 and SH-SY5Y cells (Fig. 2b). It should be noted that the spectra from the cells alone (Fig. S7) were subtracted from those of cells incubated with PG-1 (Fig. S8), to generate the spectra for the PG-1 conformational change (Fig. 2b). Our results reveal that there is a direct correlation between the presence of a CD spectra minima at 218 nm and the level of PG-1 cytotoxicity, indicating that the formation of secondary structure in PG-1 is a critical factor in predicting the level of PG-1 cytotoxicity.

### The magnitude of the positive charges on GFP determines the degree of membrane penetration and the level of mammalian cell cytotoxicity

It has been shown that the penetration of engineered GFPs with differences in positive charges into mammalian cells is related to the magnitude of their negative charges through sulphated peptidoglycan-mediated and actin-dependent endocytosis^35^. To compare the relationship between the penetration ability of super charged GFPs into cells and the level of PG-1 cytotoxicity in different cell types, we produced two differentially charged recombinant GFPs with theoretical surface charges of +15 and −17 (s-GFP +15–17) and +5 and −6 (s-GFP +5–6), respectively^36^ (Fig. S10). We incubated these s-GFPs with cells and determined the amount of internalized GFP using whole cell fluorescence and flow cytometry.

The observed frequencies of s-GFP +15–17 positive cells, obtained from an analysis of GFP fluorescence signals, were 67 ± 4%, 44 ± 4%, 51 ± 2%, 58 ± 2%, and 4 ± 3% for the 661W, NIH-3T3, HEK293T, SH-SY5Y, and 3D4/2 cells, respectively (S11), indicating significant differences existed among the different cells.

**Figure 3:**
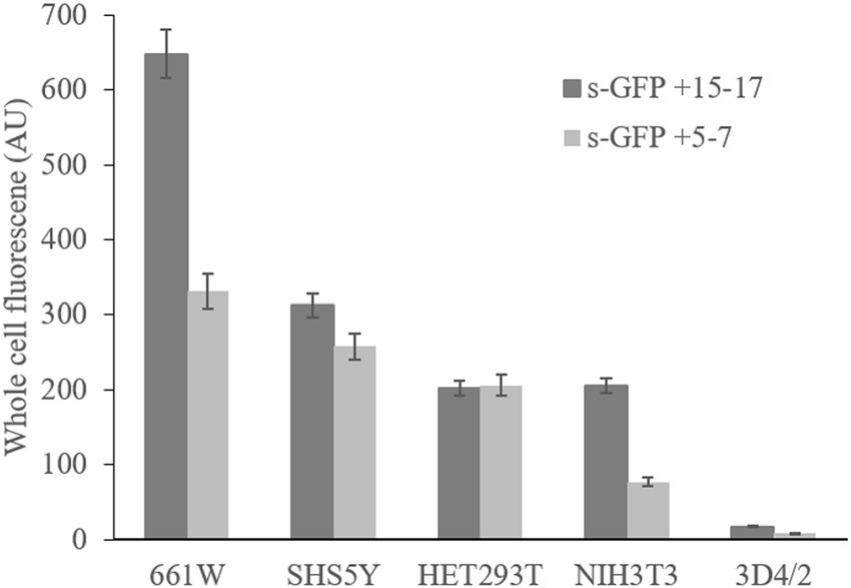
Estimation of s-GFP internalization into mammalian cells by measuring whole cell fluorescence. Whole cell fluorescence was recorded for NIH-3T3, 661W, SH-SY5Y, HEK293T, and 3D4/2 cells to estimate the internalization of s-GFP +15–17 and s-GFP +5–7, respectively. Treatments were performed using 1 μM of each s-GFP proteins. The fluorescence was measured with excitation at 490 nm and emission at 512 nm. Whole cell fluorescence was determined by subtracting the background signal of cells without s-GFP treatment from the signal of cells with s-GFP internalization. The results were presented as mean ± SD from three independent experiments.

In addition, we also measured the whole cell fluorescence of penetrated s-GFPs into cells. Whole cell fluorescence was measured after normalization by subtracting the signal from the same number of cells without s-GFPs treatment. 661W cells showed the highest level of internalization with a 2-fold difference compared to other cells (Fig. 3), consistent with the degree of PG-1 cytotoxicity toward 661W cells. After 661W cells, SH-SY5Y showed the second highest degree of s-GFP +15–17 internalization. It has been shown that the negative charges on the super charged GFPs do not significantly affect their internalization into cells^35^. Therefore, we assumed that the effect of negative charges on the s-GFPs in our experiment was negligible.

While 3D4/2 cells showed a minimal level of GFP internalization, HEK293T cells showed a similar level of GFP internalization to NIH-3T3 cells (Fig. 3). This is consistent with the result from a flow cytometry analysis (Fig. S11). However, this result partially differs from their respective cytotoxicity’s in which HEK293T cells were more significantly affected by PG-1 than NIH-3T3 cells (Fig. 1a), suggesting the mechanisms underlying the penetration of s-GFP and PG-1 cytotoxicity are not entirely identical, but both seem to be promoted by negative charges on the surface of the cells. For the remainder of the cells, the degree of cytotoxicity indirectly correlated well with the degree of s-GFP internalization.

Representative images of s-GFP internalization in 661W and NIH-3T3 cells are shown in S12. s-GFP +5–7 showed a lower rate of internalization than the highly charged s-GFP +15–17 (Figs 3 and S12). This supports the idea that differences in the electric charge of the AMP molecule itself plays an important role in interacting with mammalian cells to form a functional conformation.

### A reduction in the negative charge on the cell membrane results in a decrease in the mammalian cell cytotoxicity of PG-1

**Figure 4:**
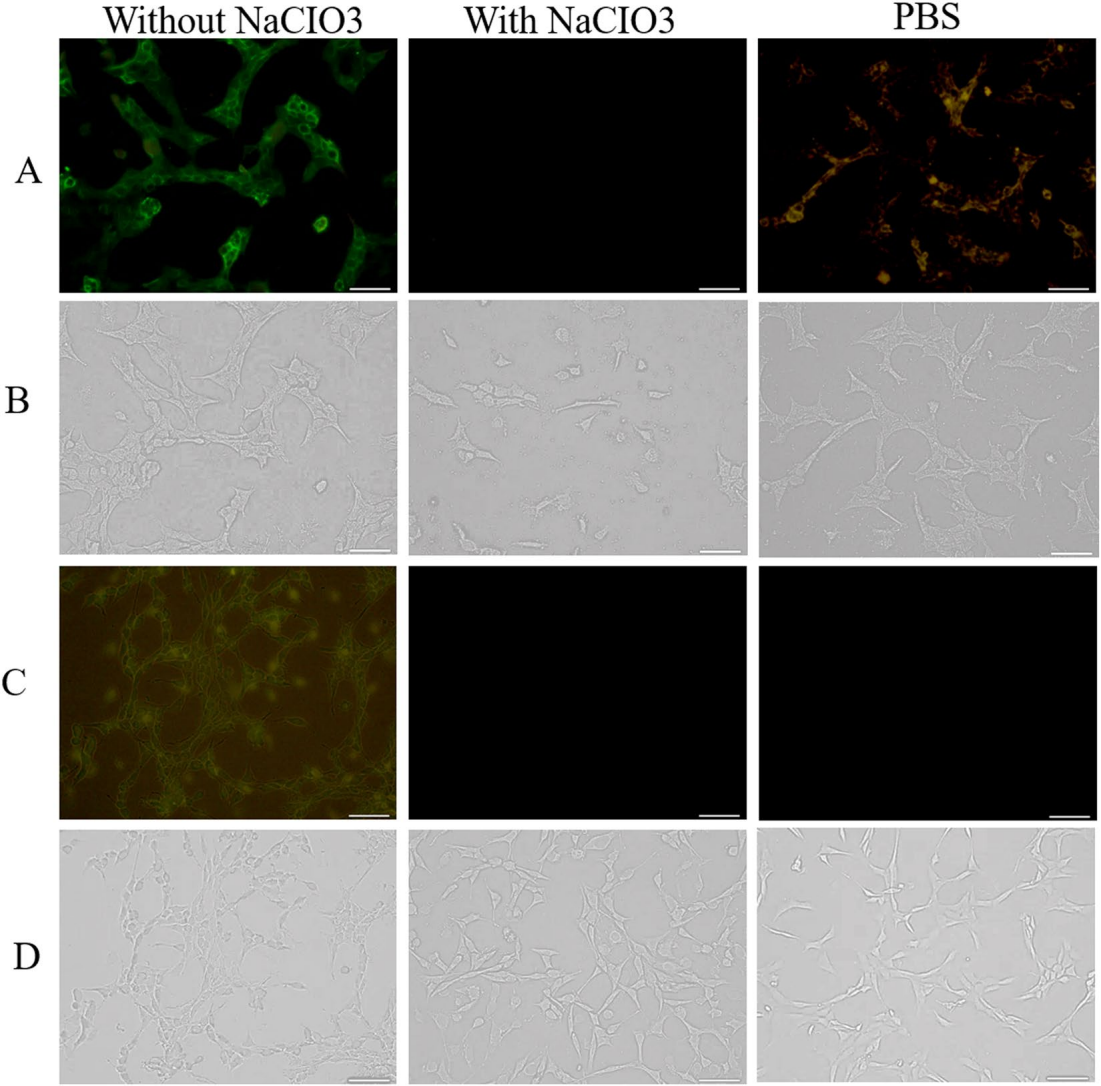
Internalization of s-GFP +15–17 into NaCIO_3_ treated and untreated 661W and NIH-3T3. 661W (**A**,**B**) and NIH-3T3 (**C**,**D**) cells were treated with and without 80 mM NaCIO_3_, respectively. These cells were also treated with s-GFPs +15–17 for 4 h. Cells without s-GFP treatment are shown labelled as PBS. The GFP fluorescent images were taken using a 424–488 nm band filter at 20x magnification (**A**,**C**) and their respective phase-contrast images are shown in panels b and d. Scale bars, 5 µm.

**Figure 5:**
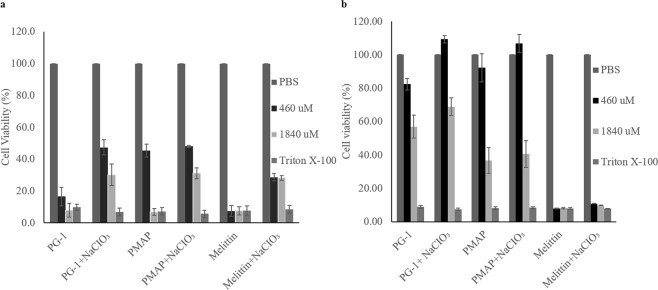
Cell viability of sodium chlorate treated 661W and NIH-3T3 cells after PG-1, PMAP-36, and melittin treatment. 661W (**a**) and NIH-3T3 (**b**) cells treated with and without 80 mM NaCIO_3_ were reacted with 2200 and 6600 μM of the AMPs; PG-1, PMAP-36, and melittin, respectively. PBS and Triton-X100 were used as negative and positive controls, respectively. Cell viability was measured after 24 h of incubation. All experiments were performed in triplicate and the standard deviations were calculated.

It has been shown that charged GFPs are able to penetrate cells through sulphated proteoglycans which are anionic in nature^35^. By treating cells with sodium chlorate, the enzyme ATP sulfurylase can be inhibited^37^ and consequently a charged s-GFP is unable to get into the cytoplasm (Fig. 4). Considering the similarity in positively charged nature between charged GFPs and antimicrobial peptides, we tried to compare the effect of a reduction in the negative charges on the surface of interacting cells using sodium chlorate treatment. In our study, s-GFP +15–17 was unable to internalize into sodium chlorate treated 661W and NIH-3T3 cells in contrast to untreated cells (Fig. 4), consistent with previous reports. We also showed that sodium chlorate treatments itself did not affect cell viability at the level (80 mM) in our experiments (S13). Next, we evaluated the changes of cytotoxicity to PG-1 in sodium chlorate treated 661W and NIH-3T3 cells. Interestingly, treatment of PG-1-treated 661W cells with sodium chlorate resulted in a significant increase in the number of viable cells compared to the same cells not treated with sodium chlorate. These results were consistent for all three AMPs, PG-1, PMAP-36, and melittin (Fig. 5a). In contrast, AMP-treated NIH-3T3 cells showed only a slight increase in the number of viable cells as a result of sodium chlorate treatment (Fig. 5b). Together, these results strongly suggest there is similarity between the internalization of positively charged GFPs and the cytotoxicity of AMPs.

### Comparison of scanning electron microscopic images between cells showing high (661W) and low (NIH-3T3) cytotoxicity to PG-1

**Figure 6:**
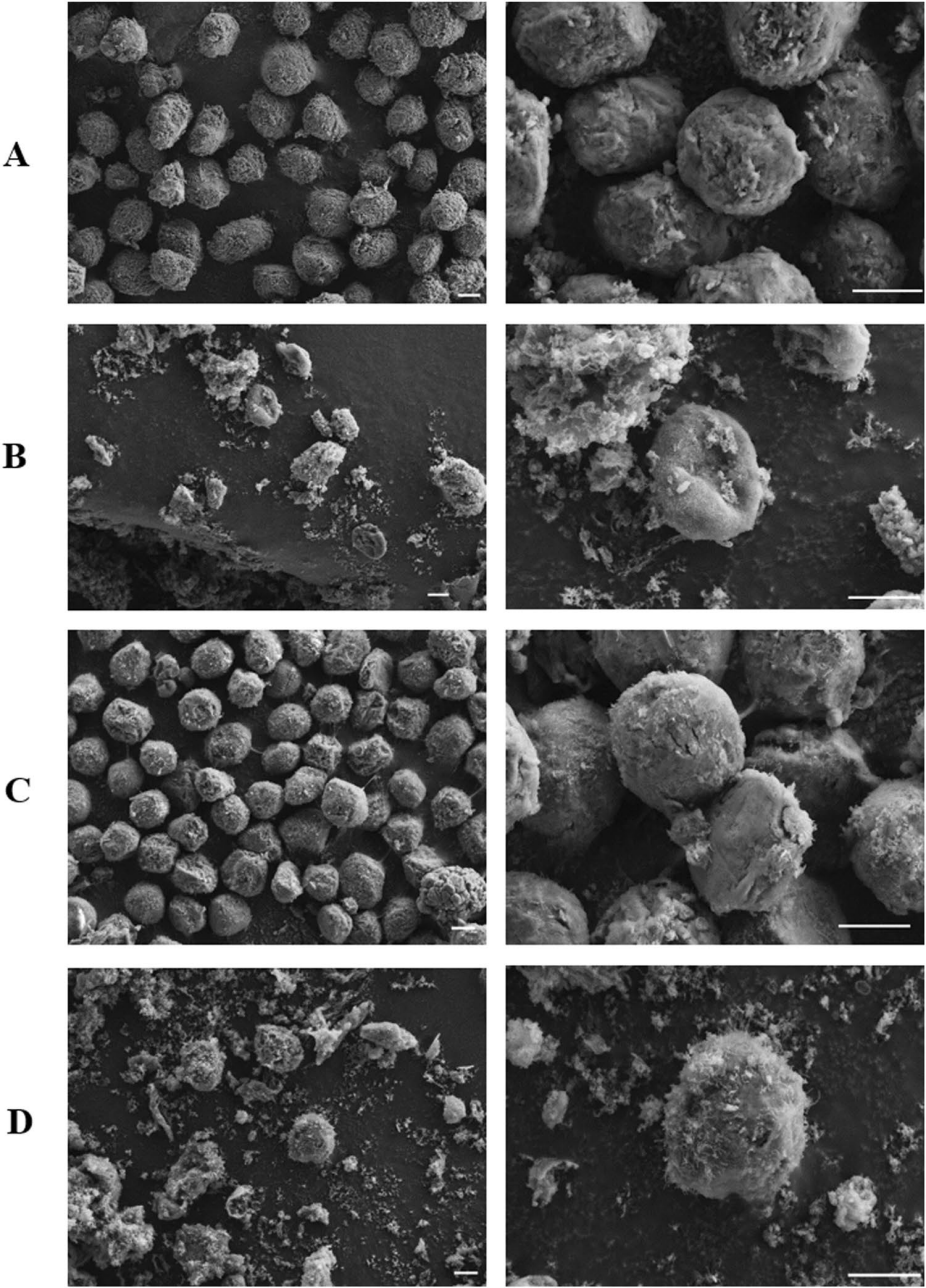
Scanning electron microscopy images of 661W and NIH-3T3 cells treated with PG-1. Electron micrographs showing the mammalian cells treated with PG-1 (panels b and d). Panels a and c are untreated controls. 661W (**A**,**B**) and NIH-3T3 (**C**,**D**) cells were incubated for 12 h with 2200 and 6600 µM of PG-1, respectively. Scale bar, 5 µm.

To visualize the morphological differences between cells with different cytotoxic susceptibilities to PG-1, we compared 661W and NIH-3T3 cells after PG-1 treatment using high resolution electron microscopy. FE-SEM images of cells after 12 h of PG-1 treatment showed that many cells were completely or partially lysed in both cell types (panel B and D) and there was a subsequent outflow of cytoplasm compared to untreated cells which had an intact morphology (panel A and C, respectively) (Fig. 6). This result was similar to the effect of PG-1 on *E. coli*^3^ and indicates that the cytotoxic effect of PG-1 in mammalian cells is also likely to be due to membrane rupturing of the treated cells and subsequent cell necrosis. However, at this resolution no clear difference was identified between these two cell types despite their different sensitivities to PG-1 cytotoxicity.

## Discussion

Previous experiments conducted in *E. coli* cells have shown that the anionic nature of their membranes attracts the cationic PG-1 to their surface, and subsequently the peptide rapidly permeabilizes the membranes resulting in cell rupture^3,4^. In addition to their bactericidal effect, AMPs such as PG-1 also shows cytotoxicity in mammalian systems^26^ which raises a concern about the use of these peptides as possible therapeutic agents. It has been reported that the mammalian cell cytotoxicity to AMPs is generally affected by factors including the degree of hydrophobicity and the magnitude of the positive charge which influences the interactions between cell membranes and AMPs^12,26,38,39^. However, the variations in their cytotoxicity toward different cell types and the mechanisms have not been well addressed.

In this study, we showed that the cytotoxic effects of PG-1 differ significantly among cell types. Using CD spectroscopy, we also showed that these differences in cytotoxicity can be explained by differences in the conformational change of PG-1 upon interacting with the different cell types. As far as we aware, this is the first report evaluating conformational changes in AMPs especially in mammalian cells using CD spectroscopy. We also showed that negatively sulphated peptidoglycans are similarly involved in the binding or penetration of positively charged GFPs and AMPs into cells.

It has been demonstrated that the degree of positive charge on a protein is important for interaction with an anionic cell surface and subsequent cell penetration, highlighting the importance of electrostatic attraction in the function of AMPs^26^. Therefore, we hypothesized that there was a possible relationship between the penetration rate of super charged GFP into cells and their sensitivity to PG-1 toxicity because of the cationic nature of cathelicidins. Blocking the formation of sulphated proteoglycans can significantly reduce affect the membranes negative charge. However, because proteoglycans are not a major component of the mammalian cell membrane, other contributing mechanisms are also be possible.

661W cells were the most susceptible to PG-1 cytotoxicity among the cells evaluated in this study (Fig. 1b,c). 661W cells lacking sulphated proteoglycans had a 3 to 4-fold increase in their overall viability (Fig. 5a). Although the rate of viability increases for NIH-3T3 cells after sodium chlorate treatment (Fig. 5b), the increase was much lower than that of 661W cells. A reason for the lower effect of sodium chlorate treatment in NIH-3T3 cells could be that they were initially less susceptible to PG-1 cytotoxicity. Similar results were obtained from the treatment of PMAP36, a porcine α-helical AMP, to 661W but no afferent differences in viability was observed from NIH-3T3. The net charges for PG-1 and PMAP36 were calculated to be +7 and +13, respectively, at physiological pH. The result from melittin (α-helix with +5) treatment was also consistent to those of PG-1 and PMAP36 although the increase of viability in sodium chlorate treated cells was lower than the others. It was reported that the analogues of cyclic gomesin showed a slight increase in cytotoxicity towards the sodium chlorate treated HL-60 cancer cells, which is the opposite to what was shown in this study^40^. The reason for such difference is unclear but it could be due to the differences in types of cells and AMPs.

Although the difference in the membrane negative charge caused by difference in sulphated proteoglycan is unlikely to be able to entirely explain variation in mammalian cell cytotoxicity to PG-1, our results suggests that charge-based interactions may be important driving forces for AMP-derived cytotoxicity because consistent results were obtained for three different AMPs against the same cell lines (Fig. 5). This suggests that sensitivity to AMP cytotoxicity could be conserved for specific cell types for different AMPs of the same subfamily.

Although we used GFPs having different charge values at the surface compared to a previous study^35^, the level of cytotoxicity seen in our cell panel could all be explained by our hypothesis with the exception of HEK293T cells (Figs 1c, 3). The lower level of charged s-GFP internalization (Fig. 3) but a shallower minimum at 218 nm in HEK293T cells compared with SH-SY5Y cells (Fig. 2b) may suggest that the surface of HEK293T cell is not highly anionic in nature compared to 661W and SH-SY5Y cells. The shallow minima CD signature might also be due to additional factors such as hydrophobicity, the amphipathic moment, or presence of bulky amino acids. Increased hydrophobicity might increase the affinity of AMPs for membranes, resulting in a higher degree of cytotoxicity^39^. Consistent with this, other β-sheet AMPs having a similar hydrophobicity to PG-1 also resulted in cytotoxicity, but PG-1 showed the highest level of cytotoxicity^18,26^.

The mechanism of action of AMPs is generally described by a few models such as the carpet, toroidal-pore, and barrel-stave models^3,41,42^. A recent study demonstrated the interaction of α-helix AMPs with intact *E. coli* membranes using CD spectroscopy^33^ and showed that the activity of AMPs depends on them adopting their proper conformation. In this study, we demonstrated the interaction of AMPs with several mammalian cells using CD. For this analysis, we used unfolded PG-1 to monitor their secondary structure formation (Fig. 2). It has been reported that the requirement for the presence of disulphide bonds is not necessarily important for the antimicrobial nor haemolytic activity of PG-1^43^, which was consistent with our results. PG-1 mutants lacking the cysteine amino acids showed antibacterial activity and formed β-hairpin like structure upon lipopolysaccharides (LPS) interactions^44^. The cys deleted mutant of PG-1 also did not show any conformational changes upon zwitterion micelles interactions. We assume that the reduced PG-1 upon interacting with different cell membranes undergoes conformational changes to form β-hairpin structure, then this may help in the formations of correct intra molecular disulfide bonds within PG-1 by oxidization of thiols. In the POPC/cholesterol membrane it was shown that β-hairpin structure was formed in the membrane surface and membrane compositions plays a decisive role^4^. Hence, the degree of β-hairpin structure formations upon different cell types (Fig. 2b) may be determined primarily due to charge and other biochemical composition of the membrane (Fig. 3).

Several theories have been proposed to explain the mammalian cytotoxicity of AMPs including non-receptor meditated interaction^26,45,46^. It has been proposed that if the hydrophobicity exceeds a threshold, then AMPs lose their membrane selectivity^45^, along with increased secondary structure propensity^22,23,45^. The binding of PG-1 to zwitterion membranes is weak but the presence of varying degree of anionic lipids in the membrane may influence the binding through electrostatic interactions^47^. In addition, a few studies have shown that the primary interaction of CAPs occurs through electrostatic interactions with anionic components in mammalian membranes due to the cationic nature of CAPs^12,38^. This may be supported by the strong shallow minima seen at 218 nm in the CD spectra seen in 661W cells and the high cytotoxicity seen in these cells in this study. Our results are consistent with a theory in which a primary initial electrostatic interaction is crucial in determining cytotoxicity. This view was also justified by a study in which cationic β-hairpin AMP SVS-1 was fully folded and was active only in the presence of a charged membrane^38^. In addition, arginine in PG-1 interacts with the phosphate atoms of lipid membranes through series of bidentate network of bonds and helps the translocation of PG-1^48^.

The higher penetration of GFP +15–17 molecules into neuronal cells (661W and SH-SY5Y cells) compared with fibroblast cells (NIH-3T3 and HEK293T) may suggest there is a higher degree of negative charge in the membrane of neuronal cells compared to other cells (Fig. 3). This could be due to the function of neuronal cells in facilitating the transport of cationic ions such as K^+^ and Ca^2+^. Our study showed that the viability of neutrophils, a main repository of AMPs, was also significantly affected by PG-1. The proprotein form of PG-1 in neutrophils may protect them from cytotoxic damage, suggesting the importance of regulatory mechanisms in AMP biology^22^. The low cytotoxicity of PG-1 toward fibroblasts and 3D4/2 macrophage cells indicates the resistance to AMP cytotoxicity among innate immune cells which frequently contact pathogens.

Not all AMPs exert severe cytotoxic effects in mammalian cells^10,11^. However, understanding the underlying mechanism allows for a window to rationally design AMPs to reduce their cytotoxicity which is a very important factor for their therapeutic application. We have shown the existence of a wide variation in cytotoxicity depending on the mammalian cell type and suggest that such variation is caused by difference in the degree of membrane negative charge. As we have shown in this study, a secondary structure analysis of AMPs in a whole cell system provides data that could model the *in vivo* situation. We feel that this is an improvement over isolated studies using LPS, micelles, or artificial membranes^4,38^. Our study should therefore contribute to deepening our understanding of the consequences of AMP treatments in mammalian systems.

## Materials and Methods

### Cell culture

NIH-3T3 (mouse embryonic cells, ATCC CRL-1658), HEK293T (human embryonic kidney cells, ATCC CRL-1573), SH-SY5Y (human neuroblastoma, ATCC CRL-2266), and 3D4/2 (porcine macrophage cells, ATCC CRL-2845), obtained from ATCC (Manassas, VA, USA), and 661W cells (mouse retinal cell)^49^ were cultured in growth media containing Dulbecco’s modified Eagle’s medium (DMEM, HyClone, GE Life Sciences, PA, USA), 10% foetal bovine serum (FBS, HyClone), 100 U/mL penicillin, 100 U/mL streptomycin, and 2 mM glutamine (HyClone). All cell cultures were maintained at 37 °C in a 5% CO_2_ humidified environment. The medium was changed every other day until the cells reached 80% confluence prior to the next passage. In addition, to inhibit the synthesis of sulphated proteoglycans in NIH-3T3 and 661W, the cells were cultured in DMEM containing 5% FBS and 80 mM sodium chlorate (NaClO_3,_ Sigma Aldrich, St. Louis, MO, USA).

### Isolation of mouse neutrophils

The experimental protocols and animal care were approved and supervised by the Institute of Animal Care and Use Committee of Konkuk University (KU15119). All animal experiments were performed and approved in accordance with the relevant guidelines and regulations set by the same committee (KU15119). All mice were maintained under specific pathogen-free conditions with unrestricted feeding. Briefly, two-month-old male BALB/6 mice were injected with 1 mL of sterile casein in 1 × PBS containing 0.5 mM MgCl_2_ and 0.9 mM CaCl_2_ (Sigma Aldrich) into their peritoneal cavity, and a second injection was carried out the following morning. Three hours later, the mice were euthanized by CO_2_ inhalation and peritoneal fluids were collected. The harvested fluid was centrifuged to collect the peritoneal exudate cells. The cells were washed three times with 1 x PBS and resuspended in 9 mL of a Percoll (GE Life Sciences) gradient solution (10 mL of 10 x PBS and 90 mL of sterile Percoll solution). The mixture was ultracentrifuged at 60000 × *g* for 20 min at 4 °C. The neutrophil-enriched (>95%) polymorphonuclear (PMN) cell layer was collected. The PMNs were washed once with 1 x PBS and resuspended in DMEM containing 15% FBS. The cells were used within 24 h of isolation.

### Cytotoxic activity assay

Cells were cultured in DMEM containing 10% FBS in 5% CO_2_ at 37 °C until they reached 80% confluence. The cells were detached by treating with accutase (Sigma Aldrich) and seeded into a 96-well plate containing 1–4 × 10^4^ cells per well in 100 µL media. The cells were treated with recombinant PG-1 (0–3680 µM), melittin (Sigma Aldrich) (460 and 1840 µM), and recombinant PMAP-36 (460 and 1840 µM) which had been produced as previously reported^31^, and the plates were incubated for 24 h in 5% CO_2_ at 37 °C. Untreated cells were used as the negative control and Triton X-100 (Sigma Aldrich) was used as the positive control for 100% cell lysis. After 24 h, the media were removed from the wells and 100 μL of fresh DMEM and 10 μL of detection reagent (Cell proliferation reagent WST-1; Sigma-Aldrich) was added to each well according to the manufacturer’s protocol and incubated further 4 h. Subsequently, the absorbance of each well was measured at 450 and 650 nm with a microplate reader (xMark™ spectrophotometer; Bio-Rad). Cell viability was calculated using the following formula: cell viability (%) = (A_t_ − A_0_)/(A_c_ − A_0_) × 100, where A_t_ is the absorbance (at 450 nm) of the treated cells, A_0_ is the background absorbance (at 650 nm), and A_c_ is the absorbance of the negative control. Additionally, dead and live cells were counted using Trypan blue (Sigma Aldrich) staining using a haemocytometer. All experiments were carried out in triplicate. IC_50_ is the measure of the peptide concentration that inhibit 50% of cells viability after 24 h of exposure. The IC_50_ was calculated by regression analysis of corresponding dose-response sigmoidal curves fitted using GraphPad Prism (San Diego, USA).

### Circular dichroism spectroscopy

All CD spectra were recorded at 25 °C using a 1 cm quartz cell over the 195 to 260 nm range in a Jasco J-810 spectropolarimeter (Jasco, MD, USA) with data modes of 1 nm bandwidth, 1 s response time, 50 nm/min scanning speed, and four accumulations. Unfolded PG-1 was prepared by reducing the peptide with 10-fold molar mass excess of DTT to PG-1 concentration in sodium phosphate buffer pH 7.4 for 5 min at 50 °C. The sample was dialyzed against water to remove the excess DTT and lyophilised. Both unfolded and folded PG-1 were suspended in 1 x PBS and CD spectra were measured. CD data obtained from samples were subtracted with the baseline spectra of 1 x PBS. To obtain the CD spectra of unfolded PG-1 interacting with *E. coli* cells (0.1 OD) was treated with 30 µM PG-1 and incubated for 2 h and the spectra was recorded under the same conditions as previously reported^33^. In the case of mammalian cells, 2 × 10^6^ cells were washed twice with 1 x PBS, suspended in 100 µL of 1 x PBS, treated with denatured recombinant PG-1 (50, 100, and 150 µM, respectively), and incubated at 4 °C with occasional gentle mixing for different time intervals (0, 1, and 4 h). The spectra were recorded by resuspending the cells to a final volume of 400 µL with 1 x PBS. The CD spectra signal of the respective mammalian cells without PG-1 was subtracted from the PG-1 treated cells to eliminate background cell signals.

### Expression and purification of supercharged GFPs

Recombinant *E. coli* BL21 (DE3) expressing two differently charged GFP proteins (s-GFP +5–7 and s-GFP +15–17)^36^ were grown at 37 °C for 5 h, respectively, after 0.1 mM IPTG treatment at an OD_600_ of 0.6–0.8. The cells were harvested by centrifugation and lysed using a Constant flow press (Constant Systems Limited, Northants, UK). The suspension was spun at 20000 *g* for 15 min at 4 °C to collect the soluble protein fraction. Protein was purified according to method previously described^36^.

### Whole cell florescence assay using charged GFPs

NIH-3T3, 661W, HEK293T, SH-SY5Y, and 3D4/2 cells were seeded into 96-well plates (5 × 10^3^ cells/well) and incubated overnight in DMEM containing 10% FBS. Following this, the media was changed to serum-free DMEM. s-GFP +5–7 or s-GFP +15–17 were added to the wells at a final concentration of 1 μM, respectively, and incubated for 4 h at 37 °C in 5% CO_2_. Cells incubated with 1 x PBS served as the control. Finally, the excess and surface-bound cells s-GFPs were were washed out with 1 x PBS twice and cells were treated with trypsin. The detached cells were washed with 1 x PBS and 3000 cells were resuspended in 100 μL of 1 x PBS for analysis. Whole cell fluorescence measurements were recorded on a Perkin Elmer/Wallac Victor 2 Multilabel Counter (1420–011, MA, USA) by measuring the fluorescence intensity at excitation and emission wavelengths of 485 nm and 515 nm, respectively, with excitation/emission slits of 5.0 nm.

### Microscopy to assess the penetration of s-GFP +15–17 into 661W and NIH-3T3 cells

661W and NIH-3T3 cells were cultured for three additional passages in the presence of 80 mM NaClO_3,_ as described above. Subsequently, 2.5 × 10^4^ cells were seeded into chamber slides in the presence of 80 mM NaClO_3_ and further grown for 24 h. The cells were then treated with 1 µM of s-GFP +15–17 for 4 h in serum free DMEM. Cells incubated with 1 x PBS without s-GFP +15–17 served as the negative control. The media were removed and washed twice with 1 x PBS to remove excess GFPs and the cover slides were mounted. Similarly, both these cells were grown in the absence of NaCIO_3_ and treated either with 1 µM s-GFP +5–7 or s-GFP +15–17 for 4 h in serum free DMEM. Images of s-GFP penetration were acquired using a fluorescence microscope with a 428–484 nm band filter (Leica, Japan).

### Scanning electron microscopy

NIH-3T3 and 661W cells were seeded at 6.0 × 10^5^ cells per well in a 6-well plate and incubated in a humidified incubator at 37 °C and 5% CO_2_ for 24 h. The cells were treated with recombinant PG-1 (1365 and 455 μM for NIH-3T3 and 661W cells based on their sensitivity to PG-1, respectively) for 12 h. The cells were harvested by centrifugation at 1000 *g* for 5 min and then washed twice with 1 x PBS. The cells were fixed in Karnovsky’s fixative (2% paraformaldehyde, 2.5% glutaraldehyde in 0.2 M cacodylate buffer, Sigma Aldrich) overnight at 4 °C. The cells were then washed three times with 0.1 M cacodylate buffer. Subsequently, the cells were post-fixed with 1% osmium tetroxide (Sigma Aldrich) in 0.1 M cacodylate buffer at 4 °C. The samples were washed three times with 0.1 M cacodylate and dehydrated through a graded series of acetone (50%, 70%, 90%, and 100%) for 15 min each time and then twice in propylene oxide for 15 min each time. The cells were further dried with hexamethyldisilazane (Daejung Chemicals and Metals Co. Ltd., Siheung, South Korea) for 15 min. For observation, the prepared samples were sputter-coated with platinum using a Cressington sputter coater (Cressington, Watford, UK). Images were acquired using a Hitachi S-2700 Scanning Electron Microscope (Tokyo, Japan).

## Supplementary information

Protegrin-1 cytotoxicity towards mammalian cells positively correlates with the magnitude of conformational changes of the unfolded form upon cell interaction
