# Supplementary material for: Protegrin-1 cytotoxicity towards mammalian cells positively correlates with the magnitude of conformational changes of the unfolded form upon cell interaction
Source: Sci Rep. 2019 Aug 9;9:11569. doi: 10.1038/s41598-019-47955-2 (PMC6689069; doi:10.1038/s41598-019-47955-2)
Supplement: Supplementary file 1 — Protegrin-1 cytotoxicity towards mammalian cells positively correlates with the magnitude of conformational changes of the unfolded form upon cell interaction [file 41598_2019_47955_MOESM1_ESM.docx]

**Protegrin-1 cytotoxicity towards mammalian cells positively correlates with the magnitude of conformational changes of the unfolded form upon cell interaction**

Nagasundarapandian Soundrarajan^1*^, Suhyun Park^1*^, Quy Van Chanh Le^1^, Hye-sun Cho^1^, Govindan Raghunathan^2^, Byeongyong Ahn^1^, Hyuk Song^1^, Jin-Hoi Kim^1^, and Chankyu Park^1#^

^1^Department of Stem Cell and Regenerative Biotechnology, Konkuk University, Gwangjin-gu, Seoul, South Korea.

^2^Department of Chemistry, University of Konstanz, Universitӓtsstraße 10, 78457 Konstanz, Germany

^*^These authors contributed equally

^#^To whom correspondence should be addressed:

Chankyu Park

Department of Stem Cell and Regenerative Biotechnology, Konkuk University

Gwangjin-gu, Seoul 05029, Korea

Tel: 82-2-450-3697/ Fax: 82-2-457-8488

E-mail: chankyu@konkuk.ac.kr

**Supplementary Information**

**Materials and Methods:**

**Expression and purification of PG-1**

The construct pET30b-based r5M-172PG1173 plasmid (Fig. S1) was transformed into *E. coli* BL21 cells and the encoded recombinant PG-1 was expressed. The recombinant protein was purified according to previously described method^1^. Briefly, target protein was expressed as insoluble fractions and purified using Ni-NTA according to manufactured protocol using FPLC (GE life sciences, PA, USA). Target protein was cleaved by cyanogen bromide (Sigma Aldrich, St. Louis, USA) to release the PG-1 from GFP scaffold (Fig. S1). Finally, PG-1 was purified from this mixture using C18 DeltaPak 7.8 ID column (Waters, MA, USA) using RP-HPLC. Purified peptide was confirmed by probing with rabbit anti-PG-1 antibody as described previously^2^. Target peptide was suspended in 20 mM sodium phosphate buffer pH 7.4 containing 8 M urea, 5 mM reduced glutathione and 0.5 mM oxidized glutathione (Sigma Aldrich) to install disulfide bond. The mixture was dialyzed exhaustively against deionized water and peptides was lyophilized. Final purity was determined by RP-HPLC using the C18 column in AKTA Pure (GE life sciences) by detecting at UV 280 nm. Briefly, the purified peptide was suspended in HPLC grade water containing 0.1% trifluoracetic acid (Sigma Aldrich), injected into C18 column and eluted using the linear gradient with 100 % acetonitrile containing 0.1% trifluoracetic acid. Target peak area was subtracted by other background peaks using Unicorn 6.3 (GE life sciences) to determine the purity. The final purity of the PG-1 was more than 95 %.

**Estimation of thiols in reduced PG-1**

PG-1 suspended in 50 mM sodium phosphate buffer pH 7.4 was reduced using DTT with minimum 10 molar mass excess to the peptide concentration used. The samples were heated for 5 min at 50°C. One portion of the mixture alone was treated with 2X concentration of iodoacetamide (Sigma Aldrich) to DTT concentration used for an additional 5 min at 50°C. Both the mixtures were dialyzed separately against water to remove the excess DTT and iodoacetamide. These reduced peptides (50 to 150 µM) were suspended in 50 mM sodium phosphate buffer pH 7.4 containing 2 mM EDTA and treated with 4 mM monobromobiamine (mBBr) (Sigma Aldrich) at 4°C for overnight in dark. Similarly, blank was prepared without PG-1 and the value was subtracted to reduce any background fluorescence and this method was modified as previously reported^3-4^. Entire experiment was carried out in N_2_ atmosphere. The derivatized samples were read in Perkin Elmer/Wallac Victor 2 Multilabel Counter (1420-011, MA, USA) with excitation at 390 nm and collected the emission at 490 nm with 5 nm slit. Since, iodoacetamide will prevent the oxidation of reduced thiols. The reduced PG-1 treated with iodoacetamide have 100 % reduced PG-1 molecules was compared with reduced PG-1 without iodoacetamide. All the experiments were carried with three replicates independently and results presented with mean ±SD.

**Evaluation of the antimicrobial activity of recombinant PG-1**

The minimal inhibitory concentration (MIC) of PG-1 was determined using a Microbial Viability Assay Kit-WST 317 (Dojindo, Japan) as previously described^4^. Briefly, the denatured PG-1 was prepared by reducing disulphide bonds by adding 20 mM DTT in 50 mM Tris pH 8.0. The protein solution was then dialyzed extensively against distilled water to remove the DTT. The protein solution was lyophilized and suspended in 1 x phosphate buffered saline (PBS) pH 7 before use. The ATCC reference strain *E. coli* 25922 was used to test the antimicrobial activity of recombinant PG-1, both denatured and folded.

**Flow cytometry**

Cells were plated into 6-well plates (5 × 10^5^ cells/well) and incubated overnight in DMEM containing 10% FBS. Twelve hours later, the medium was changed to serum-free DMEM and 1 μM s-GFP +15-17 was added to the wells. Subsequently, the plates were further incubated for 4 h at 37℃ with 5% CO_2_. Following this, the cells were washed twice with 1 x PBS and treated with trypsin at 37℃ for 5 min to remove surface-bound proteins. The detached cells were resuspended in 1 mL of 1 x PBS for analysis. The fluorescence of the internalized s-GFP+15-17 was measured and recorded by flow cytometry (FACScalibur, BD Biosciences, San Jose, CA, USA). Cell-free media and cells incubated without s-GFP were used as controls.

**S1**. Schematic of the PG1 expression construct, r5M-172-PG1-173

**
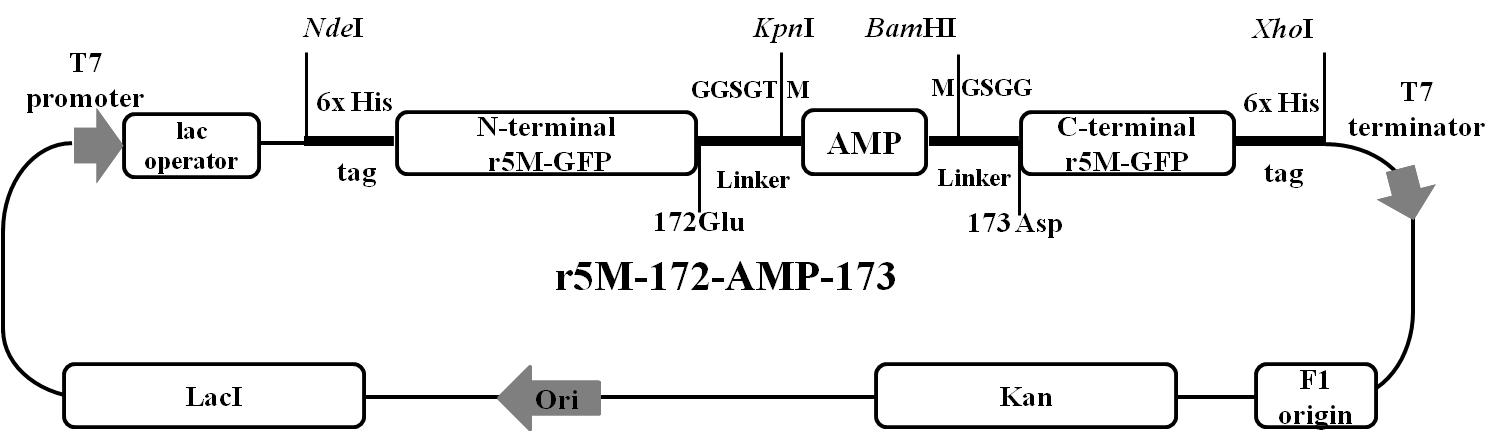
**

**S2.** Tris-Tricine SDS-PAGE analysis of purified recombinant PG-1. markers. (a) Coomassie blue-stained acrylamide gel showing the purified PG-1 indicated by the arrow. M, size marker for molecular weights. (b) Western blot analysis using a rabbit anti-PG-1 antibody. The PG-1 specific band was indicated by the arrow.


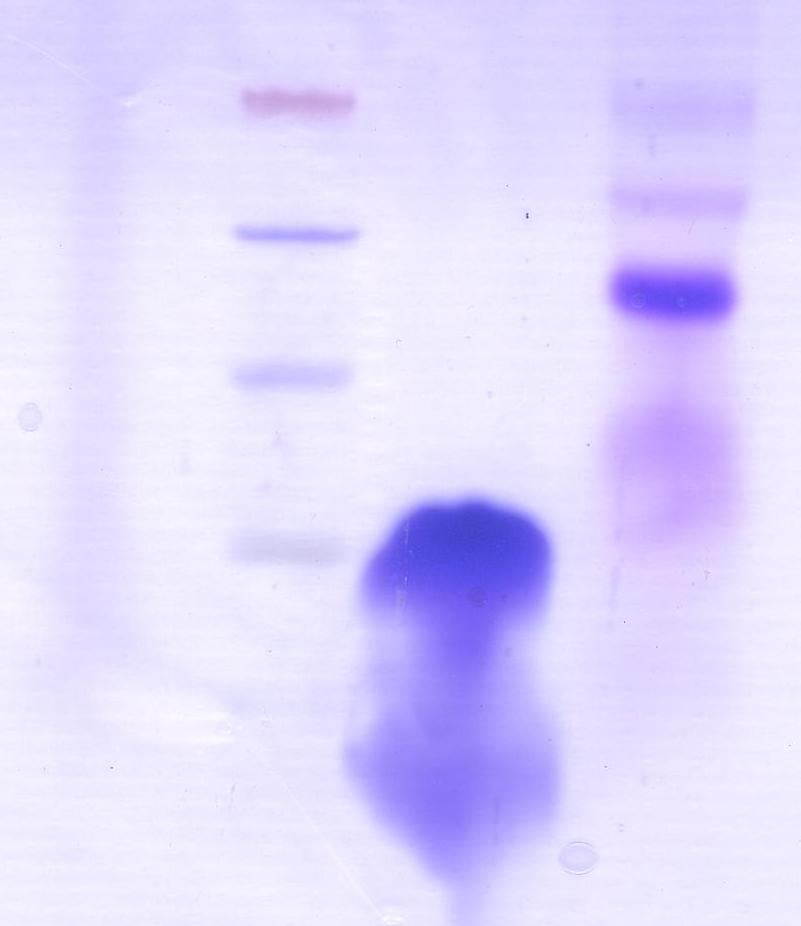


**M purified PG-1**

8 kDa

25 kDa

40 kDa

1.7 kDa

2.2 kDa


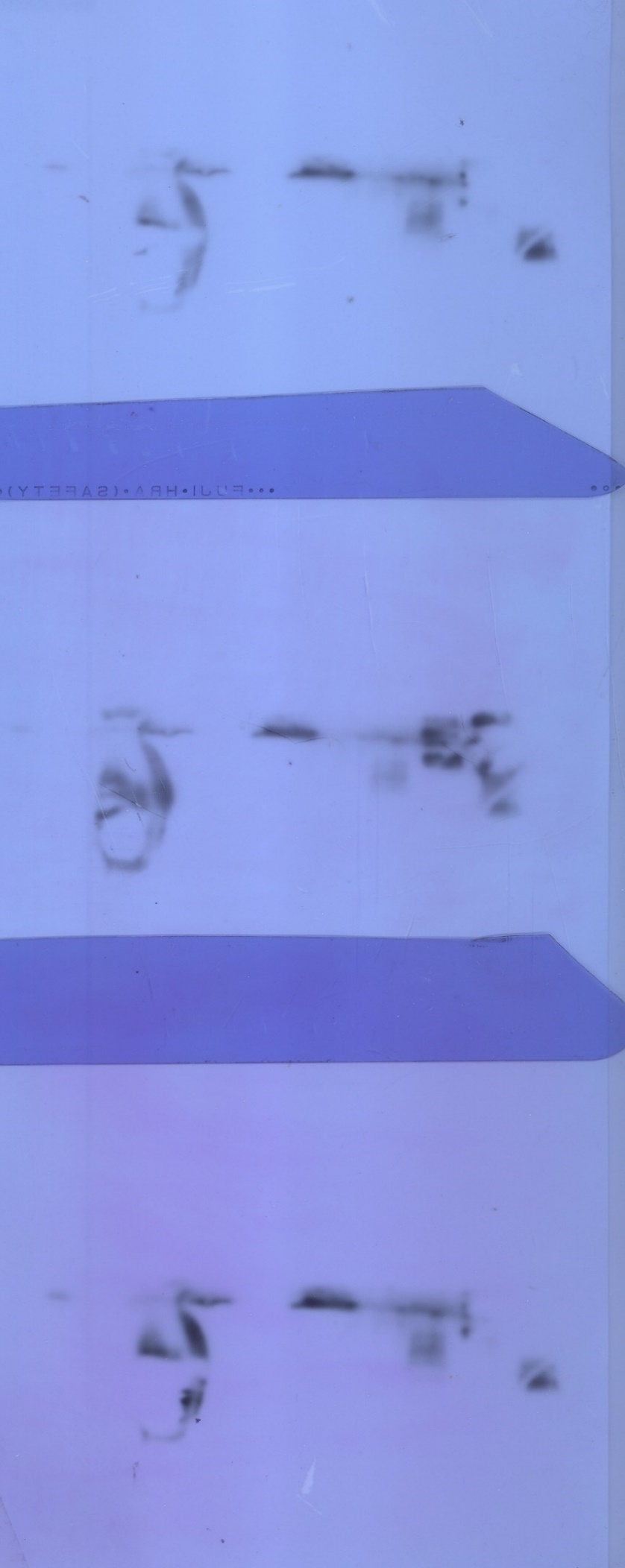


1.7 kDa

2.2 kDa

8 kDa

**a**

**b**

**S3.** Determination of the purity of recombinant PG-1. The PG-1 peptide peak was detected using UV 280 nm with a linear gradient of acetonitrile.


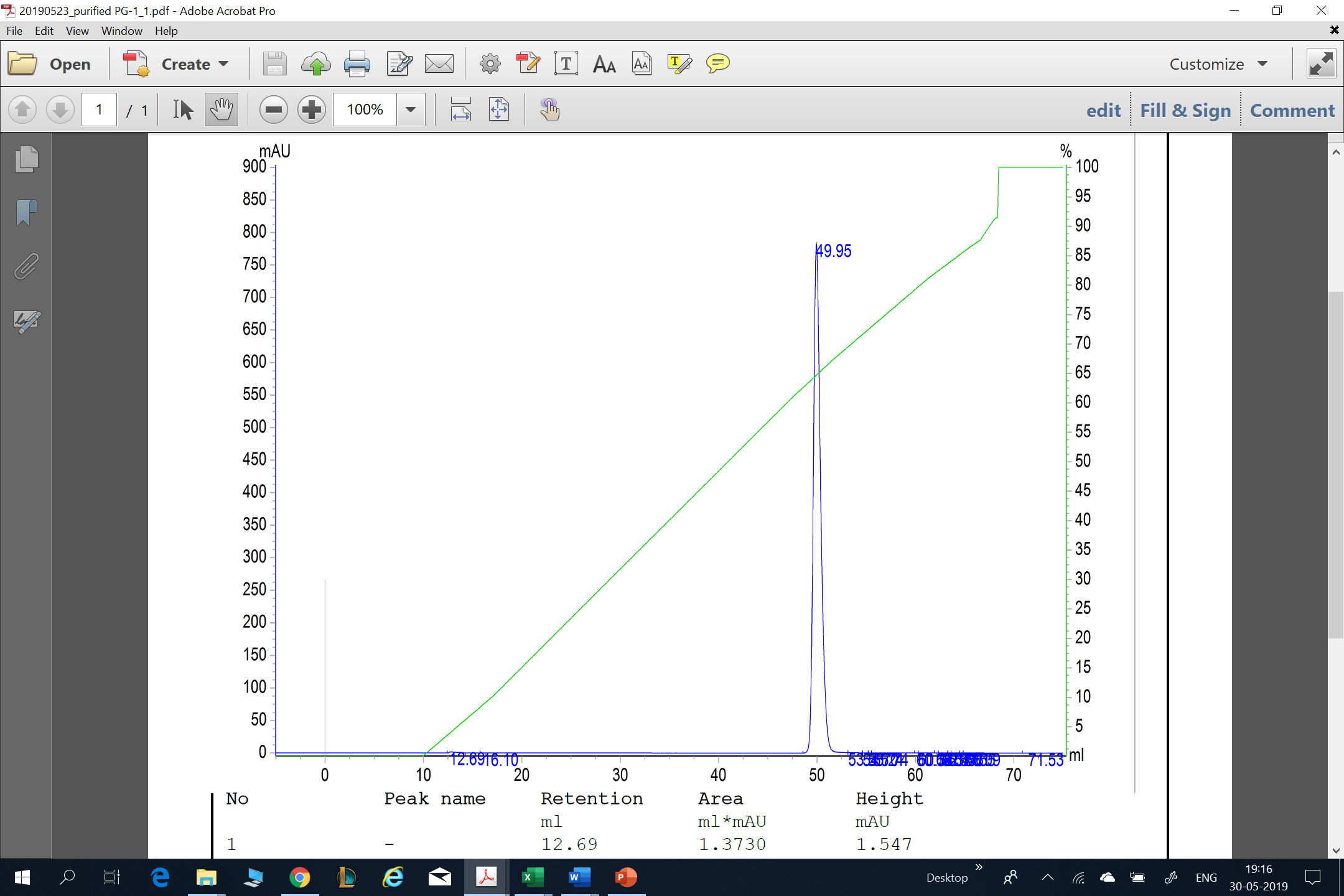


**S4.** Estimation of reduced PG-1 by DTT using monobromobiamine. All experiments were performed in triplicates and the results were shown as mean ±standard deviations.

**S5**. CD spectra of 661W cells incubated with different concentrations of PG-1 (50, 100, and 150 µM) for 1 h at 4℃. The spectra were subtracted from their respective cell spectra without PG-1.

**S6**. CD signals at 218 nm at different time points for the different mammalian cell types incubated with PG-1. The signals were subtracted from their respective cells signal at 218 nm without PG-1. Standard deviations from triplicate experiments are shown.

**S7**. CD spectra of different types of mammalian cells incubated in 1 x PBS for 4 h at 4℃.

**S8**. CD spectra of different types of mammalian cells incubated with PG-1 in 1 x PBS for 4 h at 4℃.

**S9**. CD spectra of different mammalian cells incubated with PG-1 in 1 x PBS for 1 h at 4℃. CD spectra were obtained by subtraction of the CD spectra from their respective mammalian cells without PG-1 treatment.

**S10**. SDS-PAGE analysis of purified s-GFP+15-17 (lane 1), s-GFP+5-7 (lane 2), and molecular weight markers (lane M). Arrows indicate the expected sizes of the target proteins.

**kDa**

**120**

**72**

**100**

**50**

**35**

**25**

**4**

**15**

**20**

**M 1 2**


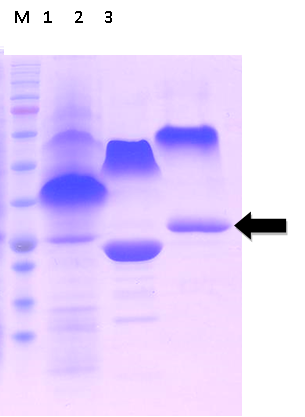


**S11: Frequency of cells showing s-GFP+15-17 internalization from a flow cytometry analysis.** The fluorescence intensity was monitored after treating various mammalian cells with 1 μM s-GFP +15-17. (A) NIH-3T3, (B) 661W, (C) HEK293T, (D) SH-SY5Y, and (E) 3D4/2 cells. GFP fluorescence was measured by excitation at 490 and emission at 520 nm. Flow cytometry analysis showing the amount of charged s-GFP internalized (green) compared to the non-internalized cells (purple) using a comparison of the ratio of mean values.

**B. 661W**

1. **NIH-3T3**


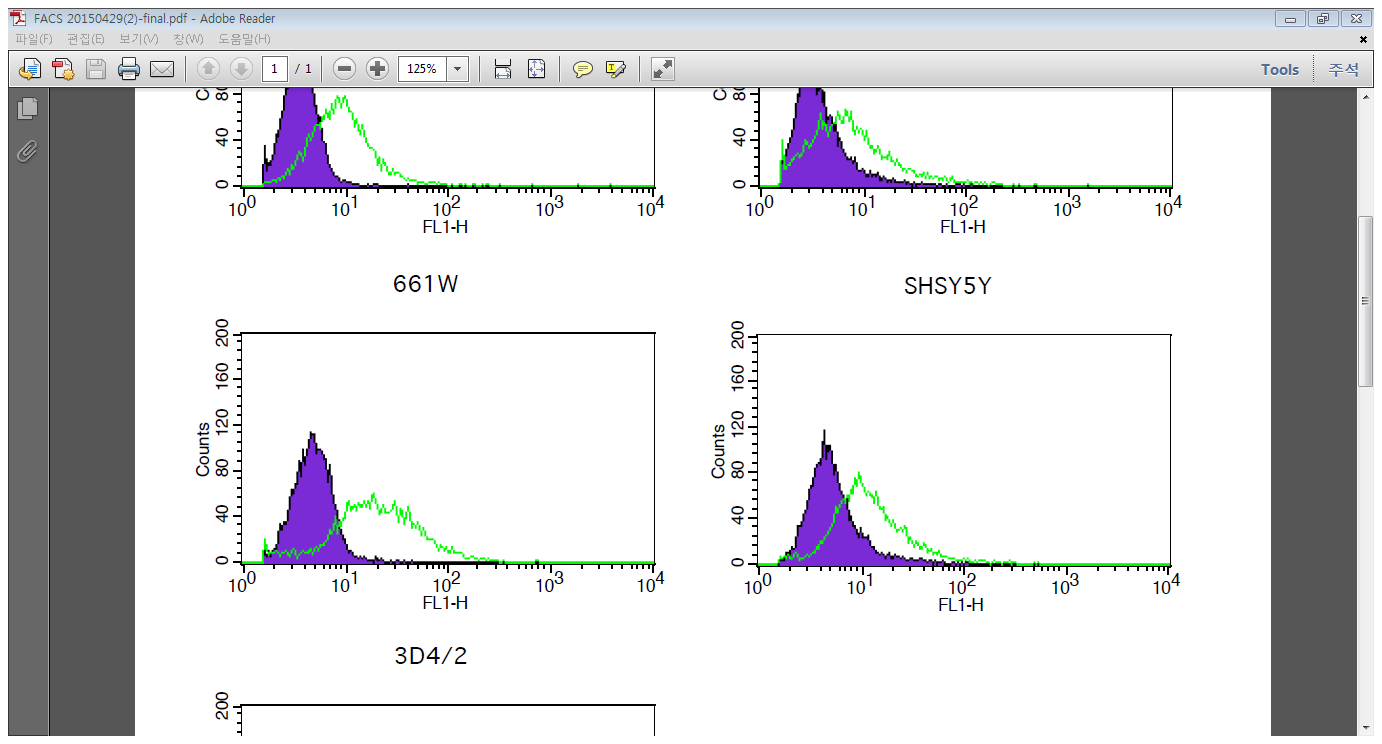

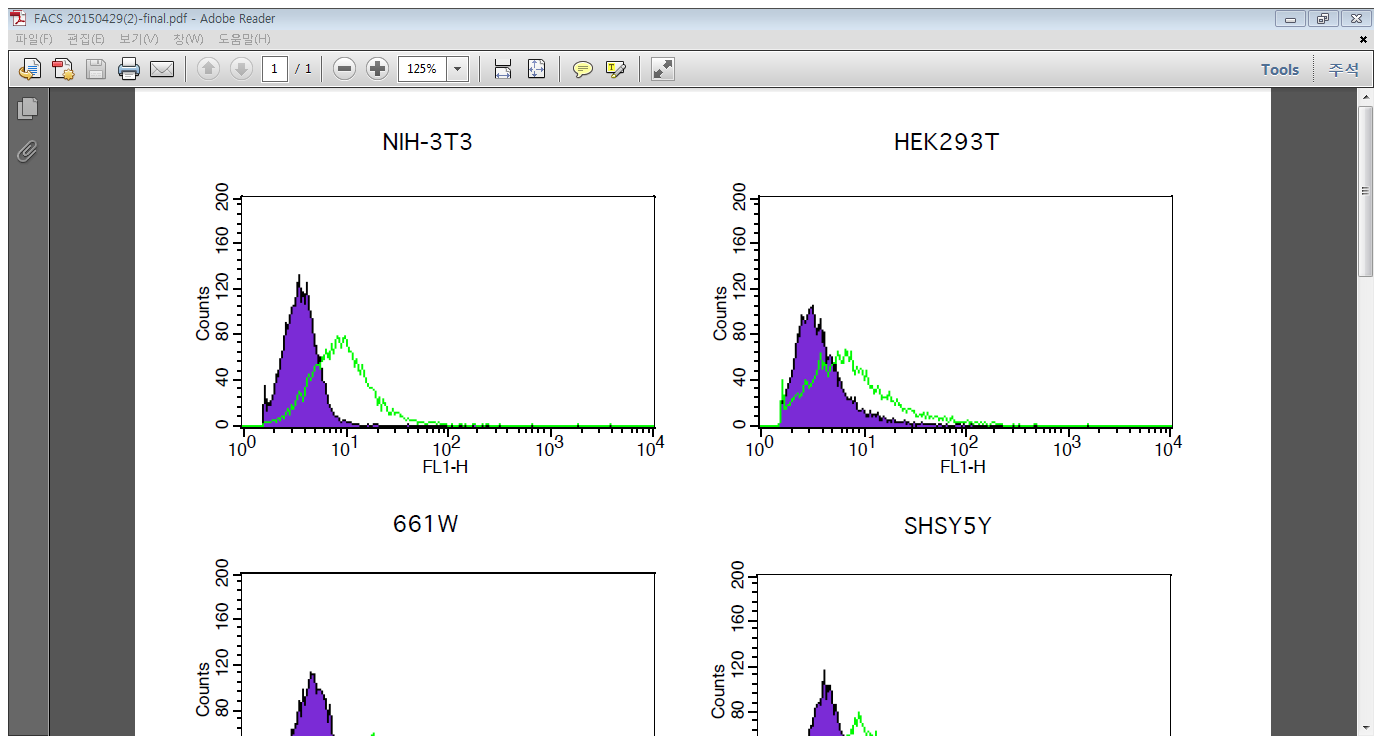


**D. SH-SY5Y**

**C. HEK293T**


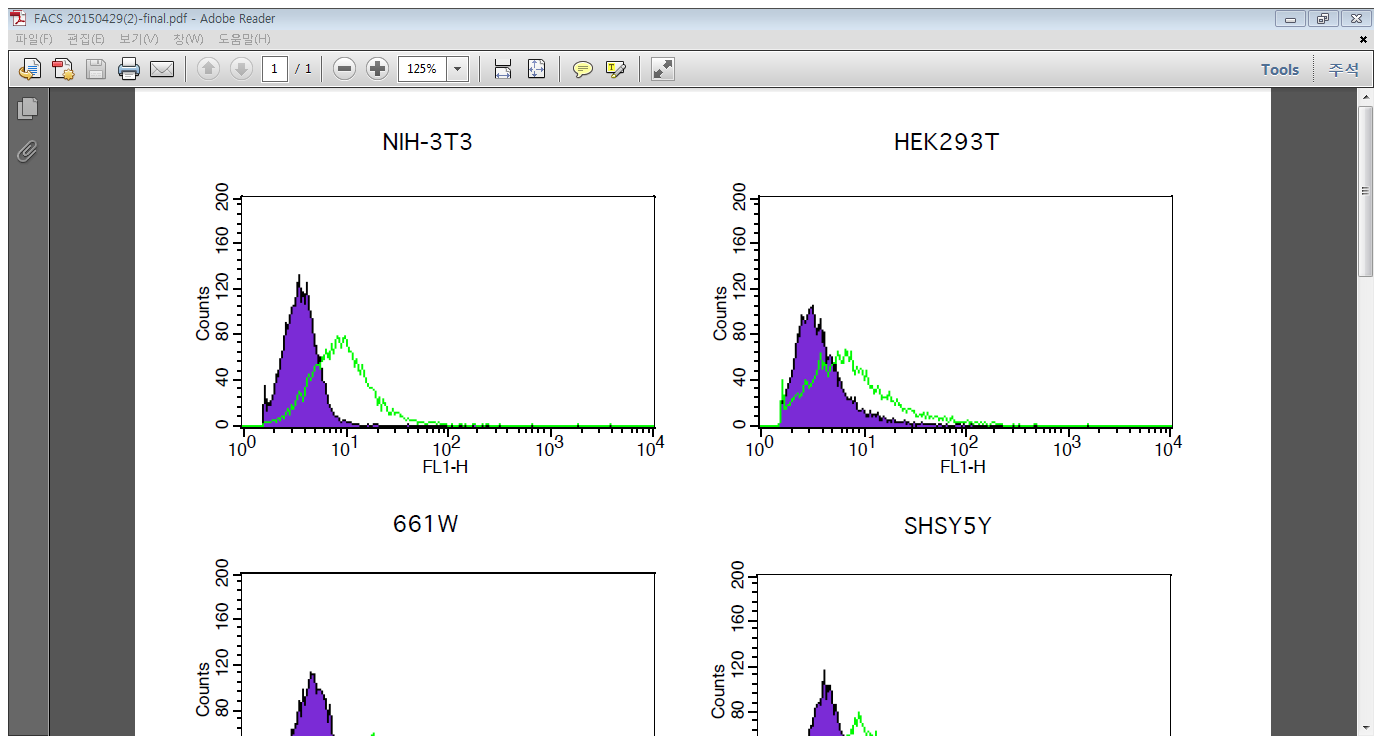

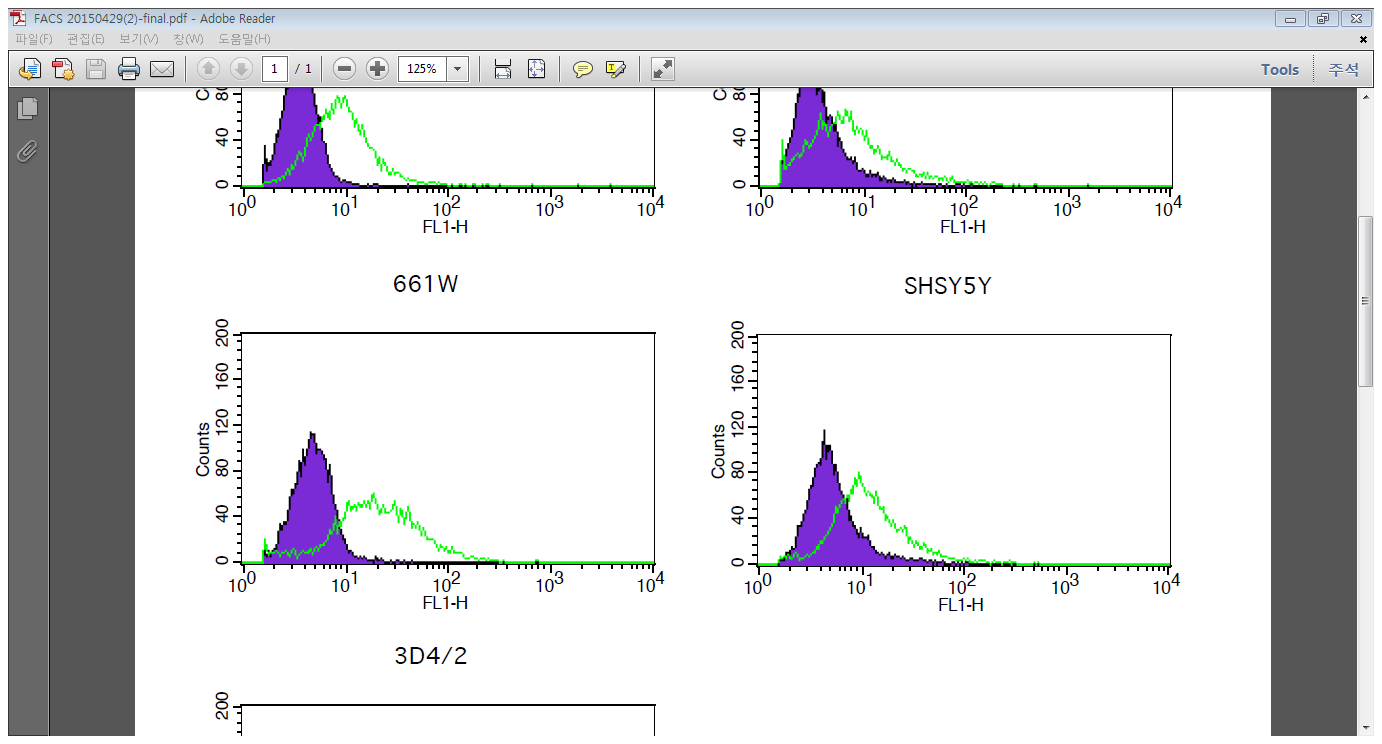


**E. 3D4/2**


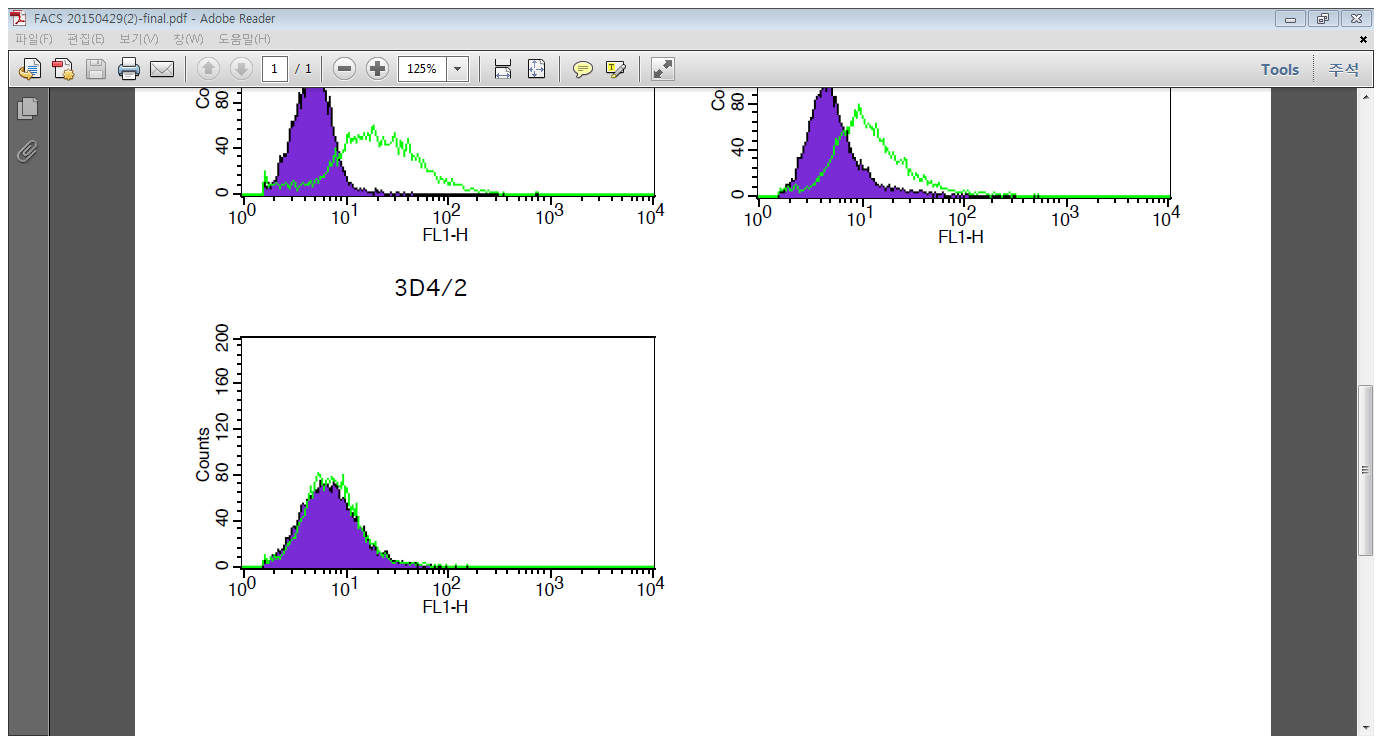


**S12: Analysis of GFP +15-17 and s-GFP +5-7 internalization into 661W and NIH-3T3 cells using fluorescence microscopy.** 661W (panel A & B) and NIH-3T3 (panel C & D) cells were treated with the different s-GFPs for 4 h. PBS was used as a negative control. Images were obtained using a 424-488 nm band pass filter (Panel A & C) with 20 x magnification and the corresponding phase-contrast pictures are shown in panels B and D, respectively. Scale bar, 5 µm.


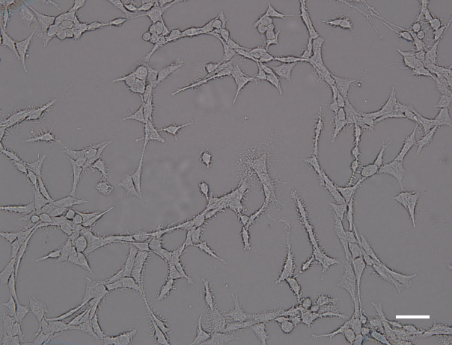

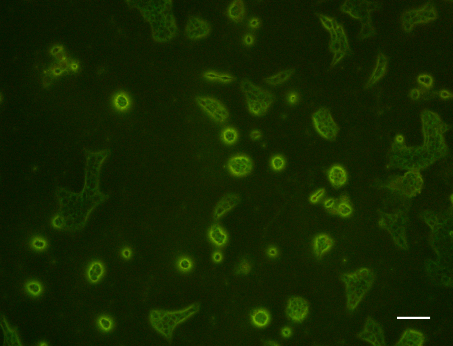

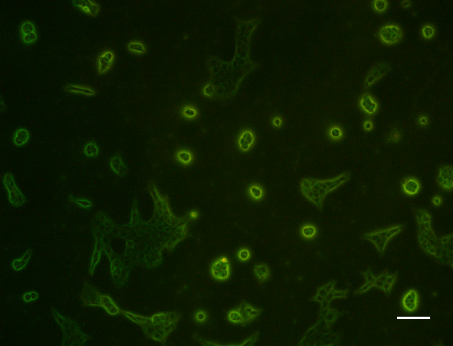

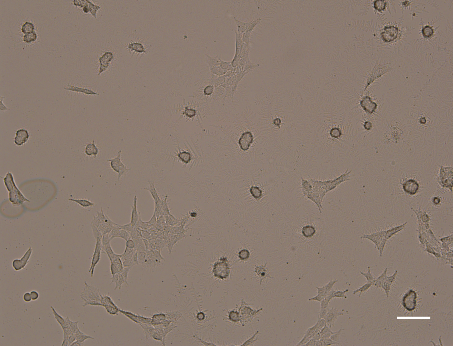

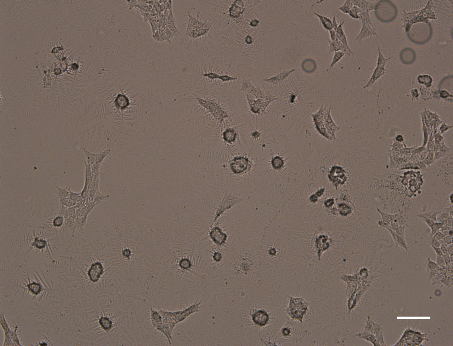

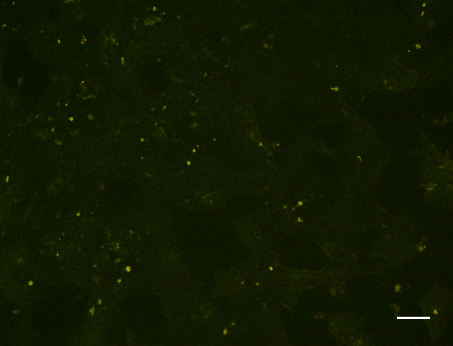

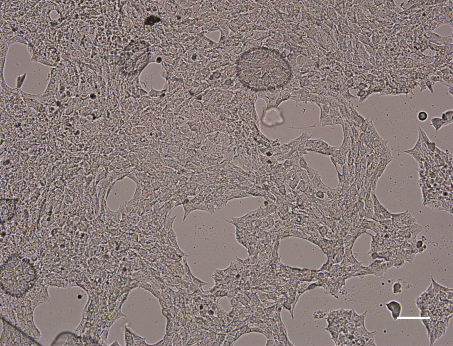


**s-GFP +15-17**


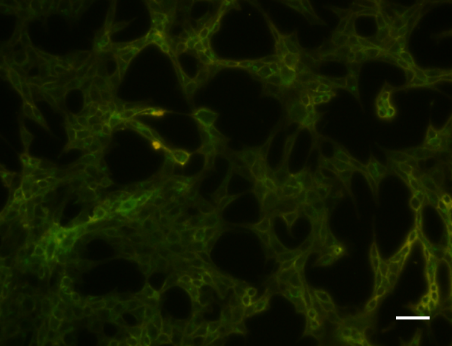

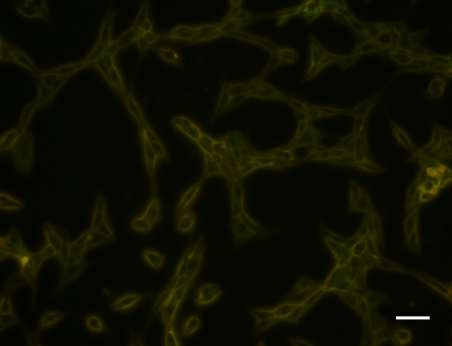

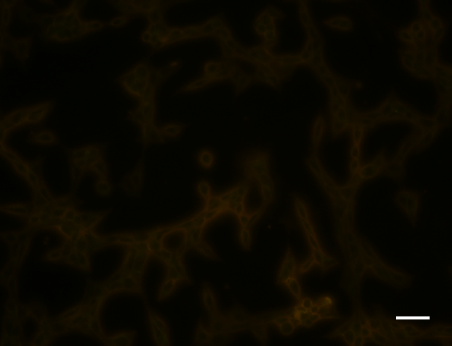


**s-GFP +5-7**

**PBS**

**A**

**D**

**C**

**B**


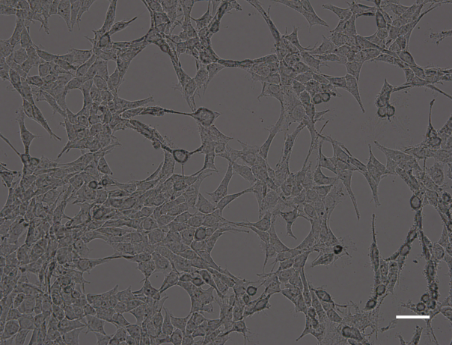

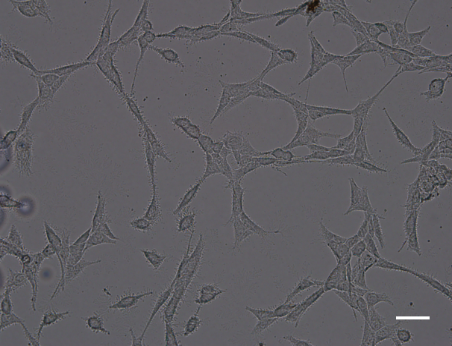


**S13.** Cell viability of 661W and NIH-3T3 cells treated with sodium chlorate. Both cells were treated with NaCIO_3_ (60 to 120 mM). PBS and Triton-X100 were used as negative and positive controls, respectively. Cell viability was measured after 24 h of incubation. All experiments were performed in triplicate and the results were mean ±standard deviations.

**ST1**. The number of intact cells from trypan blue staining. The cytotoxicity of PG-1 toward 661W cells at different time intervals (4 and 8 h). Cell counting was performed using a haemocytometer. The results were from triplicate of experiments.

| Incubation time | Number of unstained cells | | |
| --- | --- | --- | --- |
|  | PBS | PG-1 (6600 µM) | Triton X-100 |
| 4 h | 48000±522 | 4000±56 | 0 |
| 8 h | 56000±319 | 0 | 0 |

**Reference**

1. Soundrarajan, N. *et al.* Green fluorescent protein as a scaffold for high efficiency production of functional bacteriotoxic proteins in Escherichia coli. *Scientific Reports* **6**, 20661, doi:10.1038/srep20661 (2016).
2. Choi, M.-K. *et al.* Defining the genetic relationship of protegrin-related sequences and the in vivo expression of protegrins. *The FEBS Journal* **281**, 5420-5431, doi:doi:10.1111/febs.13072 (2014).
3. Fahey, R. C. & Newton, G. L. Determination of low-molecular-weight thiols using monobromobimane fluorescent labeling and high-performance liquid chromatography. *Methods Enzymol* **143,** 85-96 (1987).
4. Yang, C.S., Chou, S.T.,Liu, L., Tsai, P.J., & Kuo, J.S. Effect of ageing on human plasma glutathione concentrations as determined by high-performance liquid chromatography with fluorimetric detection. *J. Chromatogr. B: Biomed. Appl*. **674**, 23-30 (1995).
5. Kim, D. *et al.* Genomewide Analysis of the Antimicrobial Peptides in Python bivittatus and Characterization of Cathelicidins with Potent Antimicrobial Activity and Low Cytotoxicity. *Antimicrobial agents and chemotherapy* **61**, doi:10.1128/aac.00530-17 (2017).
